# Genotyping and Phylogenetic Position of *Trichinella spiralis* Isolates from Different Geographical Locations in China

**DOI:** 10.3389/fgene.2019.01093

**Published:** 2019-10-31

**Authors:** Xi Zhang, Lu Lu Han, Xiu Hong, Peng Jiang, Yui Fei Niu, Zhong Quan Wang, Jing Cui

**Affiliations:** Department of Parasitology, School of Basic Medical Sciences, Zhengzhou University, Zhengzhou, China

**Keywords:** *Trichinella spiralis*, microsatellite, genetic variance, population, China

## Abstract

In China, the nematode *Trichinella spiralis* is the main aetiological agent of human trichinellosis. We performed multi-locus microsatellite typing of *T. spiralis* isolates to improve the current knowledge of the evolution and population diversity. First, seven polymorphic microsatellite loci were used to infer the genetic diversity of *T. spiralis* collected in 10 endemic regions. Then, a Bayesian model-based STRUCTURE analysis, a clustering based on the neighbor-joining method, and a principal coordinate analysis (PCA) were performed to identify the genetic structure. Finally, the phylogenetic position of Chinese isolates was explored based on six mitochondrial and nuclear genetic markers (*cox*1, *cyt*b, 5S ISR, ESV, ITS1, and 18S rDNA) using the maximum likelihood and Bayesian methods. In addition, the divergence time was estimated with multiple genes using an uncorrelated log-normal relaxed molecular-clock model. A total of 16 alleles were detected in 2,310 individuals (1,650 muscle larvae and 660 adult worms) using seven loci. The STRUCTURE analysis indicated that the *T. spiralis* isolates could be organized and derived from the admixture of two ancestral clusters, which was also substantiated through the clustering analysis based on the allelic data. PCA separated most samples from Tiandong, Guangxi (GX-td), and Linzhi, Tibet (Tibet-lz), from the remaining isolates. However, both maximum likelihood and Bayesian inference supported the close relationship between Xiangfan, Hubei (HB-xf), and GX-td. The molecular dating analysis suggested that the Chinese isolates started to diverge during the Late Pleistocene (0.69 Mya). Generally, *T. spiralis* was observed to harbor low genetic variation, and further investigation with deeper sampling is needed to elucidate the population structure.

## Introduction

Nematodes of the genus *Trichinella* are cosmopolitan zoonotic parasites, and more than 100 species of mammals, birds, and reptiles have been confirmed as suitable hosts (Pozio, 2014). *Trichinella spiralis*, which was first described in 1881 in pork in Xiamen City, China (Manson, 1881), is the main aetiological agent of human trichinellosis, which is mainly acquired through the consumption of raw or undercooked meat infected with its larvae (Pozio, 2014). Trichinellosis has been documented in 55 countries around the world (Murrell and Pozio, 2011). In China, human cases have been recorded in 17 out of the 34 provinces/autonomous regions/municipalities (Cui et al., 2011). Until now trichinellosis has remained a serious public health problem in China.

Knowledge regarding the extent of intraspecific genetic diversity of *T. spiralis* is not only useful for understanding the dynamics of individual infections but also valuable for illustrating determinants to zoonotic risk (La Rosa et al., 2012; Zhang et al., 2015; Zarlenga et al., 2016; Zhang et al., 2017). Although great achievements have been made in the taxonomy, phylogeny, and biogeography of the *Trichinella* genus during the last decade (Zarlenga et al., 2006; Pozio et al., 2009; Mohandas et al., 2014; Korhonen et al., 2016), only a few studies have focused on the genetic diversity of the Chinese *T. spiralis* population. As described above, human trichinellosis cases have been recorded in 17 provinces/autonomous regions/municipalities of China; only seven geographical isolates from the endemic regions were identified (Yang et al., 2008; Fu et al., 2009; Wang et al., 2012; Zhang et al., 2016). Furthermore, all of the previous studies performed the genetic analysis of different *Trichinella* isolates either by few or even single molecular marker, or with insufficient sample size. Therefore, our knowledge of the population diversity of *T. spiralis* obtained from different geographical areas of China is still fragmented.

Herein, we intended to explore the genetic diversity and population structure of *T. spiralis* isolates in mainland China based on microsatellite sequences. Microsatellite DNA loci have been verified as suitable markers for inferring genetic variance and population differences in *Trichinella* species (Rosenthal et al., 2008; La Rosa et al., 2012; Garbarino et al., 2017; La Rosa et al., 2018). In addition, we performed a phylogenetic analysis using both mitochondrial (mt)-DNA, cytochrome *c* oxidase subunit I (*cox*1) and cytochrome *b* (*cyt*b), and nuclear DNA, 5S ribosomal DNA intergenic spacer region (5S ISR), ribosomal expansion segment V (ESV), internal transcribed sequence 1 (ITS1), and small subunit of nuclear ribosomal RNA (18S rRNA), to explore the phylogenetic position of Chinese isolates in the genus *Trichinella*. These markers were chosen for their usefulness in inferring phylogenetic relationships among *Trichinella* species (Zarlenga et al., 2006; Krivokapich et al., 2008; Erster et al., 2016; Zhang et al., 2018).

## Materials and Methods

### Ethics Statement

This study was conducted in accordance with the National Guidelines for Experimental Animal Welfare and the Ministry of Science and Technology of People’s Republic of China, 2006. All procedures of animal experiments in this study were approved by the Life Science Ethics Committee of Zhengzhou University (no. 2017-0118).

### *T. spiralis* Collection

**Figure 1:**
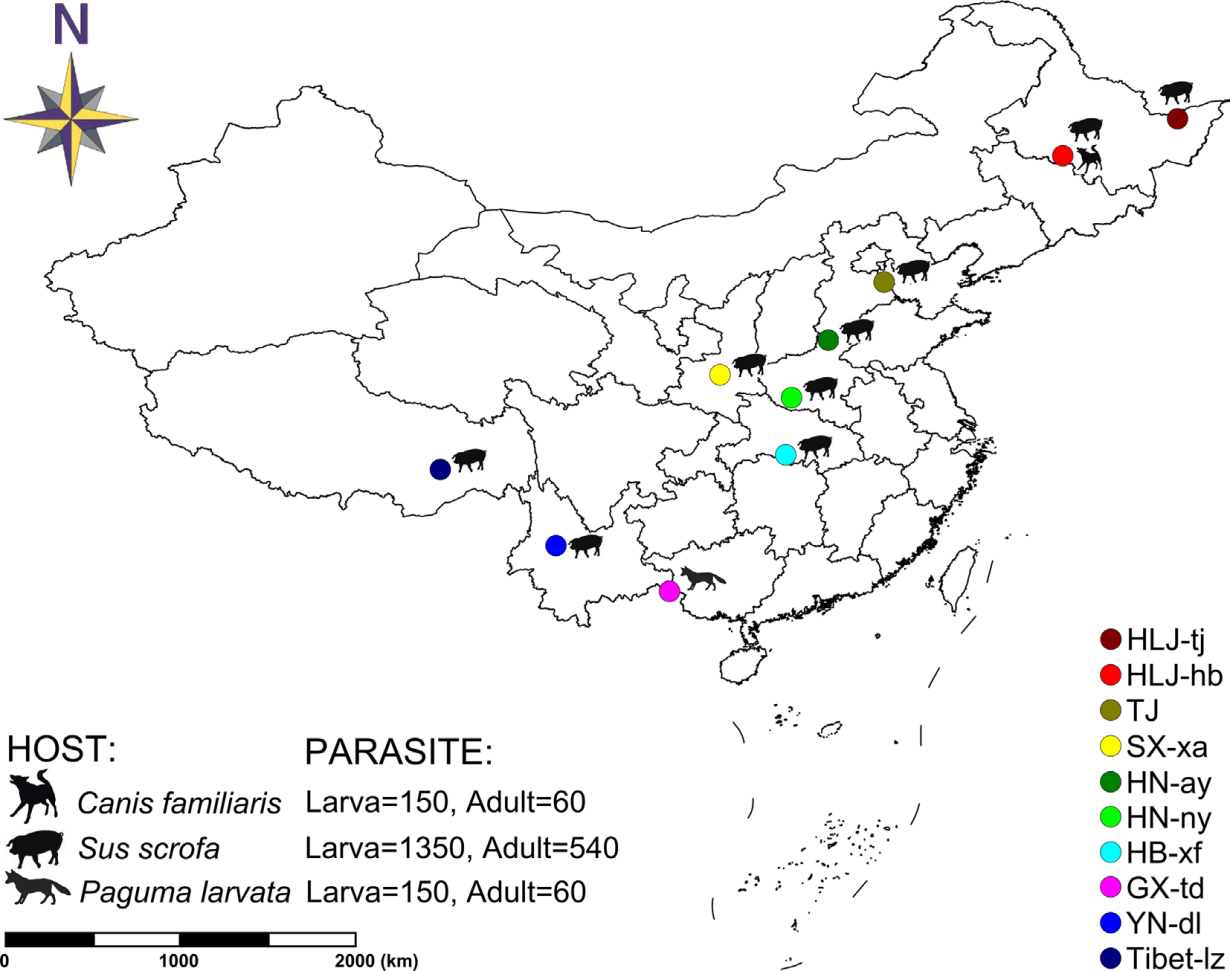
Map of collection localities and host information for *Trichinella spiralis* isolates. Geographic regions in China are designated as follows: Tongjiang, Heilongjiang (*HLJ-tj*), Harbin, Heilongjiang (*HLJ-hb*), Tianjin (*TJ*), Xian, Shaanxi (*SX-xa*), Anyang, Henan (*HN-ay*), Nanyang, Henan (*HN-ny*), Xiangfan, Hubei (*HB-xf*), Tiandong, Guangxi (*GX-td*), Dali, Yunnan (*YN-dl*), and Linzhi, Tibet (*Tibet-lz*).

In total, 11 *T. spiralis* isolates were originally collected from naturally infected hosts in 10 geographical locations in China (two isolates were collected in the sampling site of Harbin: one sample isolated from *Sus domesticus* and the other from *Canis familiaris*) (**Figure 1**). Each geographical isolate was maintained by serial passage in BALB/c mice every 6–8 months in our laboratory. To explore the genetic diversity, each geographical isolate was represented with six samples: three muscle larvae samples and three adult worm samples. And these six samples were collected from six different infected mice, three for collecting muscle larvae and three for collecting adult worms. For each sample, a panel of worms were used for DNA extraction according to the sampling method described in La Rosa et al. (2012): 50 individuals were collected for muscle larvae and 20 individuals were collected for adult worms. Finally, 2,310 individuals (1,650 muscle larvae [11 × 3 × 50] and 660 adult worms [11 × 3 × 20]) originating from 10 geographic regions were genotyped at seven microsatellite loci. Muscle larva and adult worm were separately identified to check whether or not the genetic difference is in existence during different stages of *T. spiralis*. Muscle larvae were obtained by conventional artificial digestion and stored at −80°C for later analysis (Gamble et al., 2000). Adult worms were collected from the small intestine of the infected mouse according to the methods of Sun et al. (2015).

### DNA Extraction and Microsatellite Study

For all isolates, total genomic DNA was extracted from a group of worms collected from a single infected mouse (50 for muscle larvae and 20 for adult worms) using the EasyPure Genomic DNA Kit (Transgen, China) following the manufacturer’s protocol. DNA quality was checked by agarose gel electrophoresis, and the concentration was measured with a NanoDrop spectrophotometer. Seven polymorphic microsatellite loci for *T. spiralis* (**Supplementary Table S1**) were amplified using primer combinations designed by La Rosa et al. (2012). PCR products used for genotyping were separated by electrophoresis on 8% non-denaturing polyacrylamide gels with a voltage of 100 V lasting 30 min and visualized *via* silver staining.

Population genetic parameters, including the allelic richness (*A*), the observed heterozygosity (*H*_o_), and the expected heterozygosity (*H*_e_), were performed in GenePop v4.2 (Rousset, 2008). Pairwise *F*_ST_ values between populations were calculated in Arlequin v3.5 (Excoffier and Lischer, 2010) to estimate levels of genetic differentiation among populations and among the loci. Principal coordinate analysis (PCA) in GenAlEx v6.5 (Smouse et al., 2015) was used to summarize the genetic relationship among populations and among the individuals. The structure of the *T. spiralis* isolates were determined based on allelic data using STRUCTURE v2.3.4 (Hubisz et al., 2009) by the K-means partitional clustering and admixture model (Falush et al., 2003). Twenty independent runs were conducted for each *K* = 1 to 10 using a burn-in length of 10^4^ and 10^5^ replicates of Bayesian Markov chain Monte Carlo (MCMC) sampling. Average membership coefficients for the 20 simulation runs of a given *K* value were generated by CLUMPP v1.1.2 (Jakobsson and Rosenberg, 2007), and a graphical presentation of the average membership coefficients for each isolate was generated in Microsoft Excel. The most appropriate number for *K* was calculated using STRUCTURE HARVESTER, web version (Earl and vonHoldt, 2012). The allelic data was also used to ascertain the genetic relationships among the tested genotypes by cluster analysis. The data were transformed to binary mode using scores 1/0 for presence/absence of allele, respectively, as described in Aggarwal et al. (2007). The clustering based on the neighbor-joining (NJ) method was performed in PAUP*4b10.

### Sequencing and Phylogenetic Analysis

Six target molecular markers of *Trichinella* worms, viz. *cox*1, *cyt*b, 5S ISR, ESV, ITS1, and 18S rDNA, were amplified using the primers and protocols listed in **Supplementary Table S2**. These markers have been verified useful in inferring phylogenetic relationships among *Trichinella* species (Zarlenga et al., 2006; Krivokapich et al., 2008; Erster et al., 2016). Among which, *cox*1 and *cyt*b are mitochondrial genes for representing rapid evolving sequences, and 5S ISR, ESV, ITS1, and 18S rDNA are nucleus ribosomal genes for representing medium and slowly evolving sequences. Therefore, the combination of the six genes contains sufficient genetic information in the phylogenetic analysis. PCR products were purified using the EasyPure PCR Purification Kit (Transgen, China) and sequenced in both directions using an automated sequencer (ABI Prism 3730 XL DNA Analyzer; ABI Prism, Foster City, CA) at the Genwiz Company (Beijing, China). All sequences were deposited in the GenBank database with the accession numbers shown in **Supplementary Table S3**. In addition, the related sequences of the selected markers for other *Trichinella* species and genotypes were collected from GenBank to confirm the phylogenetic position of the detected Chinese genotypes (**Supplementary Table S3**). Sequence alignment was performed in MEGA v.6.06 (Tamura et al., 2013) using the default settings. The phylogenetic position of Chinese *T. spiralis* haplotypes was estimated through maximum likelihood (ML) and Bayesian inference (BI). Sequence evolution models were selected by jModelTest 2 (Darriba et al., 2012) under the Akaike information criterion. ML analysis was performed in MEGA v.6.06. Confidence in each node was assessed by boot strapping (2,000 pseudoreplicates). BI analysis was based on MrBayes v.3.2 (Ronquist et al., 2012). The analysis consisted of two runs, each with four MCMC chains running for 1 × 10^6^ generations with sampling every 100th generation. Stationarity was assessed using a convergence diagnostic. The consensus tree was drawn after removing the first 2,000 trees (20%) as the burn-in phase. The approximate divergence times were estimated for the lineages of *Trichinella* species through the software BEAST v1.8.2 (Drummond et al., 2012). The concatenated sequence alignment was analyzed using a relaxed molecular clock model. Sequence variation was partitioned into two subsets according to different genes. Gene-specific nucleotide substitution model parameters were used, with each gene allowed to evolve at a different rate. Rate variation among the branches was modeled using uncorrelated log-normal relaxed clocks. A Yule process was used for the prior tree. Posterior distributions of the parameters, including the tree, were estimated *via* MCMC sampling. Two replicate MCMC runs were performed, with the tree and parameter values sampled every 1,000 steps over a total of 1 × 10^8^ steps. The molecular evolutionary rate was fixed to 0.01 substitutions per site per million year ago (Mya) for mitochondrial markers and 0.004 substitutions per site per Mya for nuclear ribosomal genes according to Zarlenga et al. (2006).

## Results

### Genetic Diversity of *T. spiralis* Isolates

Seven polymorphic microsatellite markers were used to assess and compare the genetic variability of Chinese *T. spiralis* isolates. From each of 11 natural isolates, 150 larvae and 60 adults were genotyped at each of the seven loci with the exception of the isolate from HLJ-hb, which was genotyped with 300 larvae and 120 adults (150 larvae and 60 adults originated from the domestic pig; the others originated from the dog, as shown in **Figure 1**). The analysis showed clear genotype patterns for all worms. A total of 16 alleles were observed from 2,310 worms. With the exception of the TS103 and TS1131 loci, which identified three alleles, each locus identified only two alleles, and the average number of alleles per locus was 2.29. The overall observed heterozygosity (*H*_o_) and expected heterozygosity (*H*_e_) ranged from 0 to 1 and 0 to 0.68182, respectively. The mean *H*_e_ values were generally lower than the *H*_o_ in all 10 isolates with all seven loci (**Table 1**). The *F*_ST_ values, which are used as a measure of genetic differentiation between the populations, were very low but significantly different between HB-xf and the remaining isolates (with the exception of HN-ay, HN-ny, and GX-td), between GX-td and the remaining isolates (with the exception of HB-xf), and between Tibet-lz and the remaining isolates (**Table 2**).

**Table 1:** General features of the genetic variability of the Chinese *Trichinella spiralis* isolates tested.

Populations (*N*)	Parameters	Microsatellite loci						
		TS103	TS128	TS130	TS1122	TS1131	TS1380	TS1444
HLJ-hb (420)	*A*	2	1	1	1	2	1	1
	*H*_o_	0.91667	0	0	0	0.25000	0	0
	*H*_e_	0.51812	0	0	0	0.22826	0	0
HLJ-tj (210)	*A*	1	1	1	1	1	2	2
	*H*_o_	0	0	0	0	0	0.33333	0.16667
	*H*_e_	0	0	0	0	0	0.30303	0.16667
TJ (210)	*A*	1	1	1	1	1	2	1
	*H*_o_	0	0	0	0	0	0.16667	0
	*H*_e_	0	0	0	0	0	0.16667	0
SX-xa (210)	*A*	2	1	1	1	2	1	1
	*H*_o_	0.66667	0	0	0	0.16667	0	0
	*H*_e_	0.48485	0	0	0	0.16667	0	0
HN-ay (210)	*A*	1	2	1	1	2	1	1
	*H*_o_	0	0.33333	0	0	0.33333	0	0
	*H*_e_	0	0.30303	0	0	0.30303	0	0
HN-ny (210)	*A*	1	1	1	2	2	2	2
	*H*_o_	0	0	0	0.83333	0.16667	0.33333	0
	*H*_e_	0	0	0	0.53030	0.16667	0.30303	0.30303
HB-xf (210)	*A*	1	2	1	2	2	2	2
	*H*_o_	0	0.33333	0	0.50000	0.83333	0.16667	0
	*H*_e_	0	0.30303	0	0.40909	0.53030	0.16667	0.48485
GX-td (210)	*A*	2	2	2	2	2	1	2
	*H*_o_	0.33333	0.66667	0.66667	0.50000	1.00000	0	0
	*H*_e_	0.30303	0.48485	0.48485	0.40909	0.68182	0	0.48485
Tibet-lz (210)	*A*	2	1	1	2	2	1	1
	*H*_o_	0.66667	0	0	0.83333	0.66667	0	0
	*H*_e_	0.48485	0	0	0.53030	0.48485	0	0
YN-dl (210)	*A*	1	1	1	1	1	1	1
	*H*_o_	0	0	0	0	0	0	0
	*H*_e_	0	0	0	0	0	0	0

Nnumber of individuals analysed; A, number of alleles; H_o_, observed heterozygosity; H_e_, expected heterozygosity.

**Table 2:** Genetic differentiation (*F*_ST_) values among *Trichinella spiralis* isolates.

	HLJ-hb	HLJ-tj	TJ	SX-xa	HN-ay	HN-ny	HB-xf	GX-td	Tibet-lz	YN-dl
HLJ-hb	0.000									
HLJ-tj	0.036	0.000								
TJ	0.051	0.083	0.000							
SX-xa	0.056	0.050	0.064	0.000						
HN-ay	0.049	0.044	0.055	0.055	0.000					
HN-ny	0.030	0.080	0.064	0.041	0.043	0.000				
HB-xf	**0.068**	**0.045**	**0.068**	**0.060**	0.010	0.009	0.000			
GX-td	**0.210**	**0.139**	**0.179**	**0.165**	**0.137**	**0.096**	0.037	0.000		
Tibet-lz	**0.207**	**0.150**	**0.177**	**0.164**	**0.120**	**0.131**	**0.108**	**0.016**	0.000	
YN-dl	0.055	0.069	0.086	0.067	0.058	0.051	**0.076**	**0.206**	**0.205**	0.000

Significant comparisons (P < 0.05) are in bold.

### Genetic Structure of *T. spiralis* Isolates

**Figure 2:**
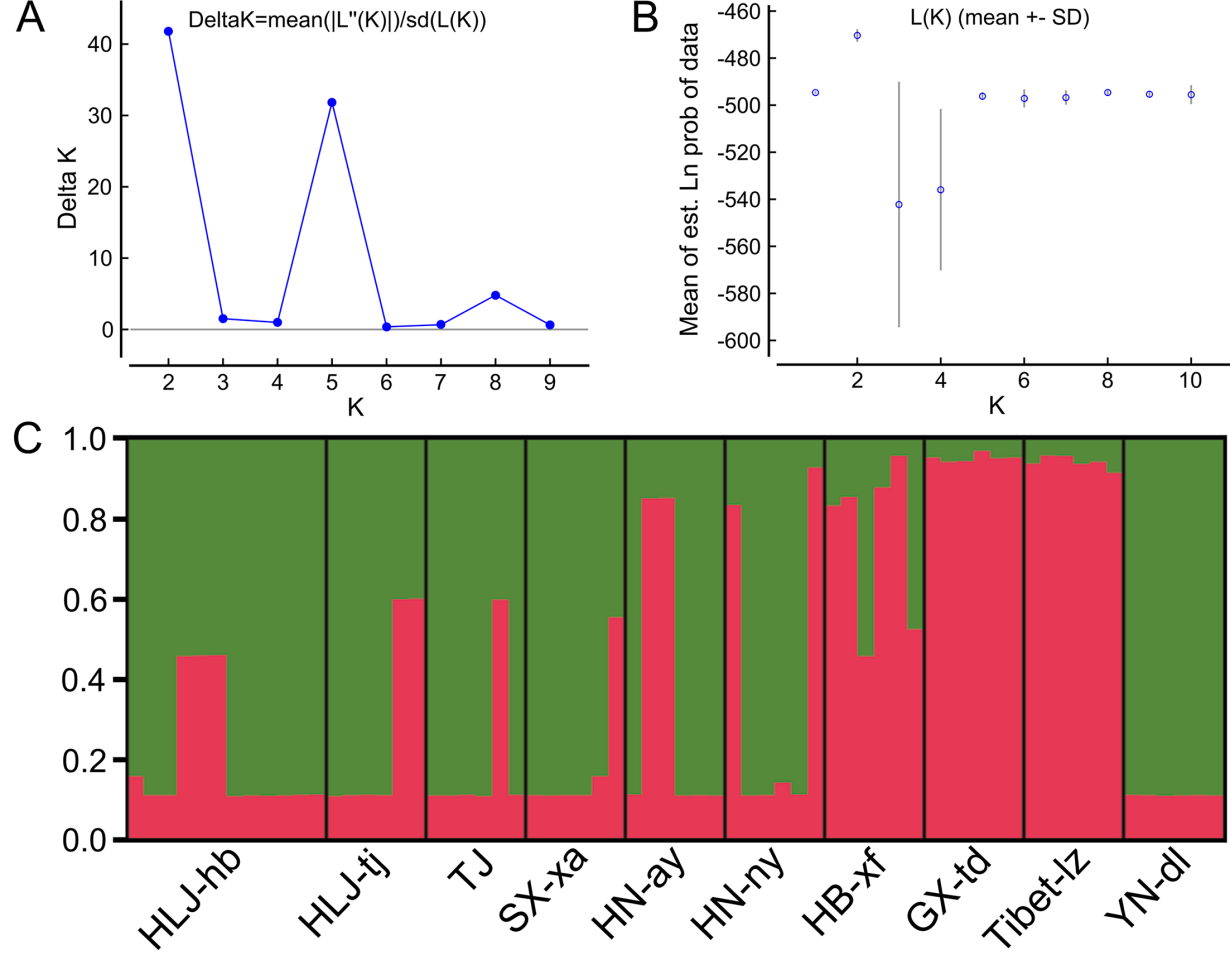
Estimated genetic structure of *Trichinella spiralis* in China as inferred by the STRUCTURE software on the basis of the data on seven microsatellite markers obtained for 770 individuals from 10 geographical locations. **(A)** Plot of the mean posterior probability (Ln*P*(*D*)) values per cluster (*K*) based on 20 replicates per *K*, generated by the STRUCTURE software, and **(B)** Delta-*K* analysis of Ln*P*(*K*). **(C)** STRUCTURE plots grouped by the Q-matrix (estimated membership coefficient for each sample) at *K* = 2. Each strain is represented by a *vertical line*, which is partitioned into the *coloured segments* that represent the parasite estimated membership fractions in *K*. The *same colour* indicates that the isolates belong to the same group. *Different colours* for the same isolate indicate the percentage of the genotype shared with each group. For the isolate code, see **Figure 1**.

**Figure 3:**
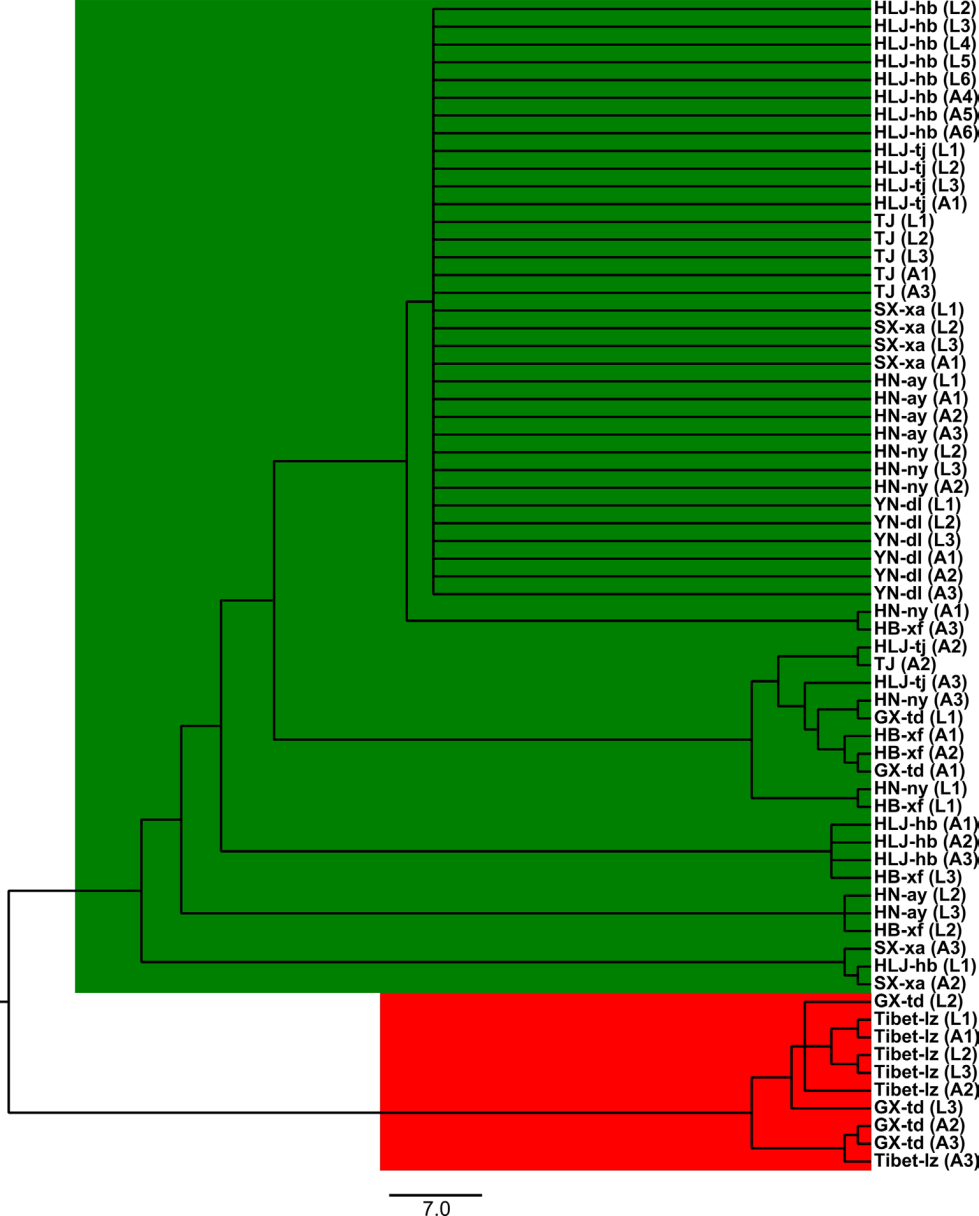
The unrooted neighbor-joining tree based on the seven microsatellite data. Within the parenthesis, “*A*” represents adult worm and “*L*” represents larva; *numbers* indicate the code of the infected mouse from which the parasite was collected. For the isolate code, see **Figure 1**.

**Figure 4:**
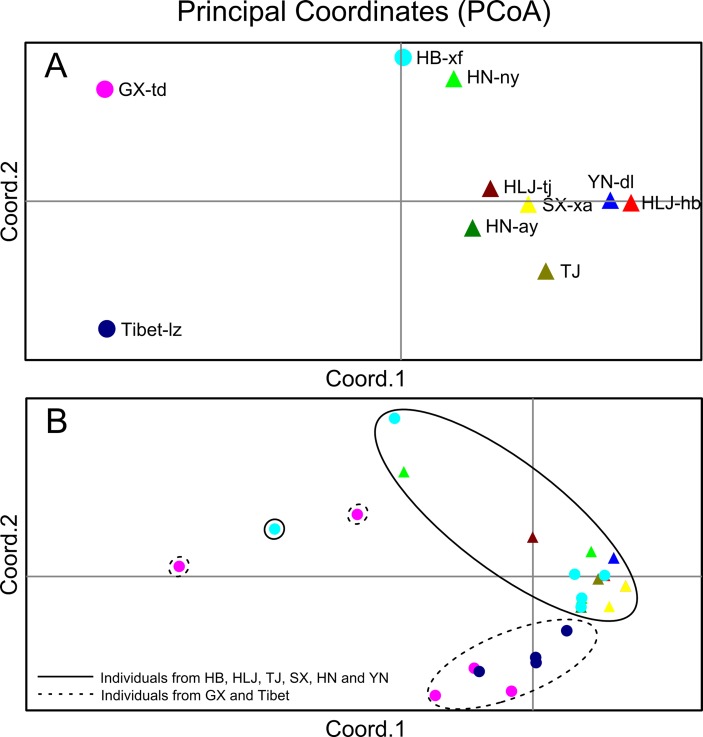
**(A)** Principal coordinate analysis (PCA) describing the relationships of the 10 *Trichinella spiralis* isolates studied based on the *F*_ST_ values calculated using seven microsatellite markers. **(B)** PCA describing the relationships of 770 single *T. spiralis* (each *point* represents a single worm) based on the covariance values calculated using seven microsatellite markers. For the isolate code, see **Figure 1**.

The Bayesian model-based clustering analysis implemented in STRUCTURE indicated that the *T. spiralis* isolates could be organized and derived from the admixture of two ancestral clusters (*K* = 2) based on the results of both the maximum Δ*K* and maximum *L*(*K*) (**Figures 2A, B**). The analysis carried out with the STRUCTURE program did not divide the 10 isolates according to their geographical origin. Samples from HLJ-hb, HLJ-tj, TJ, SX-xa, HN-ay, HN-ny, and YN-dl revealed one prominent genotype, whereas isolates from GX-td and Tibet-lz indicated the other genotype (**Figure 2C**). In consistent with the STRUCTURE analysis, the neighbor-joining tree also revealed two main clusters. As shown in the tree topology, individuals from Tibet-lz and the GX-td formed a single clade; the remaining samples made up the other one (**Figure 3**). The PCA separated HB-HLJ-TJ-SX-HN-YN isolates from those originating in GX-td and Tibet-lz (**Figure 4A**). Analyzing the genetic distances among the individuals by PCA without *a priori* assignment to specific isolates showed the following: 1) individuals from HLJ-TJ-SX-HN-YN clustered together overall with the exception that several individuals from HN-ny and HB-xf were separated further apart; 2) although partial samples from GX-td distributed separately, isolates from GX-td and Tibet-lz clustered together (**Figure 4B**).

### Phylogenetic Position

**Figure 5:**
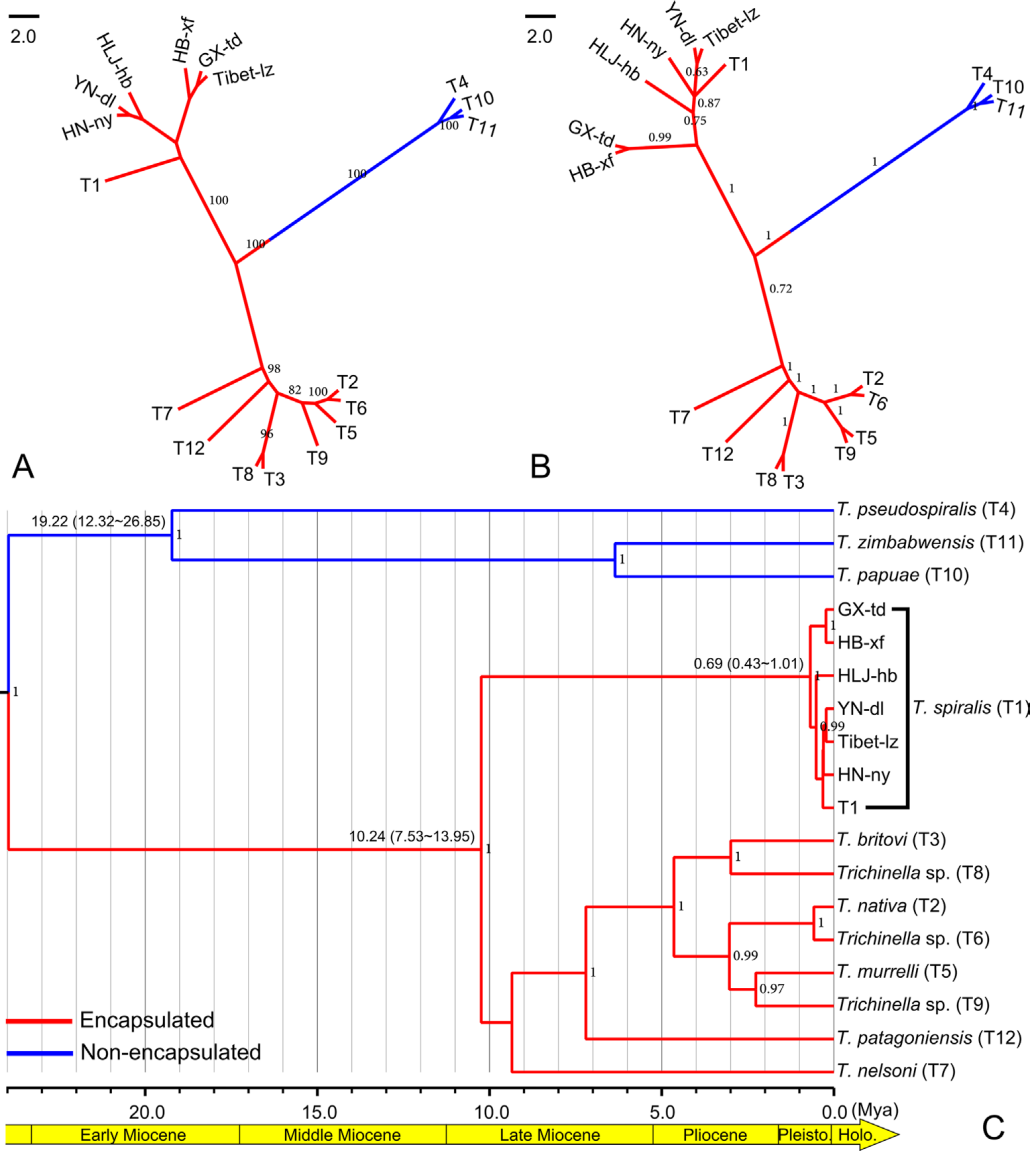
**(A)** Maximum likelihood phylogenetic tree of *Trichinella spiralis* from China. Only bootstrap values above 60 are shown. **(B)** Bayesian phylogenetic tree of *T. spiralis* from China. Only posterior probabilities values above 0.6 are shown. **(C)** BEAST chronogram of the *Trichinella* species. *Numbers in the nodes* represent the Bayesian posterior probabilities; only the posterior probabilities above 0.6 are shown. *Numbers above the branches* indicate the estimated ages for the corresponding clade with values of the 95% highest posterior density interval in the *parenthesis*.

According to the genetic structure analysis described above, we selected six isolates, HLJ-hb, HN-ny, HB-xf, and YN-dl (representing the HB-HLJ-TJ-SX-HN-YN cluster) and GX-td and Tibet-lz (representing the GX-Tibet cluster), to explore the phylogenetic position of Chinese isolates in the genus *Trichinella*. The likelihood models identified by the jModelTest suggested that the TN93 + G model was most suitable for concatenated data. In the phylogenetic analysis, for the whole genus of *Trichinella*, both the maximum likelihood and Bayesian methods generated consistent tree topologies (**Figures 5A, B**). When focused on Chinese isolates, although with low support values, ML analysis generated two separate clades: one including HLJ-hb, HN-ny, and YN-dl and the other containing GX-td, HB-xf, and Tibet-lz (**Figure 5A**). Under the BI analysis, the earliest diversifications gave rise to isolates from HB-xf and GX-td, and then to HLJ-hb. The next diversification event had separated isolates from HN-ny, YN-dl, and Tibet-lz (**Figure 5B**). However, with the exception of the HB-xf + GX-td clade, the support values for other Chinese isolates under the BI method were low. The molecular dating analysis suggested that the encapsulated lineage and the non-encapsulated lineage started to diverge in the Early Miocene (**Figure 5C**). The origin times of both lineages were estimated to be approximately 10.24 and 19.22 Mya, with a 95% highest posterior density (HPD) of 7.53–13.95 and 12.32–26.65 Mya, respectively. For the Chinese isolates, the earliest branching event was estimated to be during the Late Pleistocene (0.69 Mya, with a 95% HPD of 0.43–1.01 Mya).

## Discussion

Although a complex of at least 12 species and genotypes of the genus *Trichinella* can cause veterinary or medical problems in a broad geographic range throughout the world (Korhonen et al., 2016), only *T. spiralis* is the main aetiological agent of human trichinellosis in China, causing mild to severe clinical symptoms (Cui et al., 2011). Human cases of trichinellosis have been recorded in many parts of China. However, the genetic variability in Chinese *T. spiralis* isolates is still unknown. Here, we prepared 3,850 individuals originating from 10 geographic regions representing 10 *T. spiralis* isolates and performed a genetic variation analysis of these samples using seven microsatellite loci to investigate the genetic structure.

The genetic variability in *T. spiralis* was first described by La Rosa et al. (1992). Then, some pioneering parasitologists concentrated their studies on the genetic diversity of *Trichinella*, and many excellent advances have been made using different methods: restriction fragment length polymorphism and single-strand conformational polymorphism (RFLP-SSCP) analysis (Wu et al., 2000), “cold” single-strand conformation polymorphism analysis (Gasser et al., 2005), and deep resequencing of the mitochondrial genome (Webb and Rosenthal, 2010; Mohandas et al., 2014). In contrast to those methods based on single genes or multiple mitochondrial markers, microsatellites are distributed throughout the genome, and many are located in the non-coding parts of the genome, allowing them to accumulate mutations unhindered over the generations and giving rise to variability, which can be used for a more powerful exploration of genetic diversity (Richard et al., 2008). La Rosa et al. (2012) developed seven useful microsatellite markers for the genotyping of *T. spiralis*. In this study, we used these seven loci to identify the intraspecific variation of *T. spiralis* isolates from China, and a few interesting discoveries were made: 1) In contrast to the studies of Rosenthal et al. (2008) and La Rosa et al. (2012), which displayed relatively high genetic variations among the Asian *T. spiralis* isolates, the overall genetic diversity in the Chinese isolates was low. 2) The Bayesian model-based clustering analysis showed that the *T. spiralis* isolates could be organized and derived from the admixture of two ancestral clusters, indicating that more than two adults gave rise to the ensuing population of each geographical location. 3) The extent of genetic diversity in isolates from GX-td and Tibet-lz was higher than in the worms collected from the remaining locations. High genetic variability within GX-td isolates might be related to the host of *Paguma larvata*, as pervasive host switching plays an important role in the diversification of *Trichinella* species (Zarlenga et al., 2006). For the isolates from Tibet-lz, high diversity is probably attributable to the extreme environment in the Tibetan Plateau.

In agreement with previous studies (Yang et al., 2008; Fu et al., 2009; Wang et al., 2012), the taxonomic position of Chinese *Trichinella* belongs to *T. spiralis*, which was unambiguously supported in the phylogenetic analysis. Although the exact divergence pattern of Chinese isolates was unresolved, the ML method supported two clades, HLJ-hb + HN-ny + YN-dl and GX-td + HB-xf + Tibet-lz. The close relationship between HB-xf and GX-td was also supported in the BI analysis. The BEAST chronogram of the *Trichinella* lineage generated here suggested that the divergence between the encapsulated and non-encapsulated clades took place in the Early Miocene, earlier than that estimated in Zarlenga et al. (2006), but almost consistent with the analysis of Korhonen et al. (2016) using thousands of shared single-copy orthologous gene sequences, indicating that these markers are suitable for a molecular dating analysis of *Trichinella* organisms. Based on the molecular dating analysis, the genetic diversification of Chinese isolates was postulated to start in the Late Pleistocene, yet the exact driving force for the diversity of *T. spiralis* remains unclear. Here, we hypothesize that there are probably two driving forces in the diversification of Chinese isolates: 1) The diversification is associated with episodic periods of host switching, as ecological transitions from omnivory to facultative and obligate carnivory among mammalian hosts have been recognized in the transmission dynamics of *T. spiralis* (Zarlenga et al., 2006; Pozio et al., 2009), and 2) the diversification is associated with faunal expansion and geographic isolation during climatological and ecological perturbations in Late Pleistocene (Zarlenga et al., 2006; Hoberg and Brooks, 2008). Considering that human cases of trichinellosis have been reported in more than half of the Chinese provinces (Cui et al., 2011), the sampling locations are too few to reflect the true pattern of the genetic structure of Chinese *T. spiralis* isolates. Therefore, we need further investigation with deeper sampling (especially in the areas where there are cases of trichinellosis) to elucidate the population structure.

## Conclusions

A total of 16 alleles were detected in samples collected from 10 geographical locations using seven loci. Chinese *T. spiralis* isolates could be organized based on the admixture of two ancestral clusters. The overall genetic diversity in the Chinese isolates was low. Principal component analysis separated most samples of GX-td and Tibet-lz from the remaining isolates. Although the exact divergence pattern of Chinese isolates was unresolved, the close relationship between HB-xf and GX-td was supported in the phylogenetic analysis. The molecular dating analysis suggested that the Chinese isolates started to diverge in the Late Pleistocene.

## Data Availability Statement

Publicly available datasets were analyzed in this study. This data can be found here: GenBank Nos. MH289505-MH289540.

## Ethics Statement

The animal study was reviewed and approved by Life Science Ethics Committee of Zhengzhou University.

## Author Contributions

XZ, ZW, and JC conceived and designed the experiments. XZ, LH, XH, PJ, and YN performed the experiments. XZ and LH analyzed the data. JC, XZ, and ZW contributed reagents/materials/analysis tools. XZ, ZW, and JC wrote the paper. All authors read and approved the final version of the manuscript.

## Funding

This work was supported by the National Key Research and Development Program of China (2017YFD0501302), the National Natural Science Foundation of China (U1704284 and 81672043), the Henan Province Science and Technology Key Project (no. 182102310075), and the China Postdoctoral Science Foundation (2018T110740).

## Conflict of Interest

The authors declare that the research was conducted in the absence of any commercial or financial relationships that could be construed as a potential conflict of interest.
