# Supplementary material for: Genotyping and Phylogenetic Position of Trichinella spiralis Isolates from Different Geographical Locations in China
Source: Front Genet. 2019 Oct 31;10:1093. doi: 10.3389/fgene.2019.01093 (PMC6834790; doi:10.3389/fgene.2019.01093)
Supplement: Supplementary file 1 [file DataSheet_1.doc]

**Genotyping and phylogenetic position of** *Trichinella spiralis* **isolates from different geographical locations in China**

Xi Zhang, Lu Lu Han, Xiu Hong, Peng Jiang, Yui Fei Niu, Zhong Quan Wang*, Jing Cui*

**Supporting information**

**Table S1.** Primer sets used to amplify microsatellites identified in *Trichinella spiralis*.

**Table S2.** Primers used to amplify the sequences studied.

**Table S3.** Chinese *Trichinella spiralis* isolates and other related *Trichinella* species included in the molecular analysis, and accession numbers of the corresponding individual sequence.

**Table S1.** Primer sets used to amplify microsatellites identified in *Trichinella spiralis*.

| Locus | Primer set | Repeat motif | Reference |
| --- | --- | --- | --- |
| TS103 | ATTAAGAGGGGAGGGGGTAA | (TATT)_n_ N_74_(TTCG)_n_ | La Rosa et al. (2001) |
|  | GAATAGCTGCTAGAAGTGCCG |
| TS128 | TACATGACCGAAAAGTCGGAAA | (TTTG)_n_ |
|  | GCCCTGAGAGTAGATGAGCAAAA |  |
| TS130 | ACAATTTCAACCCACCCT | (GAT)_n_ |
|  | TCCTCATTATCGTCTTCGTC |  |
| TS1122 | GGCAACTACTACCTACCGCT | (CAC)_n_^a^ |
|  | GCTGACAATCCGATCCA |  |
| TS1131 | ACACCACCTTCACCACCA | (TAA)_n_ |
|  | TAATCACGACAAAAGATCTCCC |  |
| TS1380 | CGGACAGATTCAGCGGA | (TGC)_n_N_43_(TGA)_n_N_23_(CTG)_n_N_17_(TGA)_n_^b^ |
|  | ATGGGCCAACAACTACCACTA |
| TS1444 | CAAACGGATACACAATAGGAAG | (TTC) _n_ |
|  | AAAACAAAGGCAAGGCAC |  |

The subscript ‘n’ means a variable number of repeats; ^a^ Interrupted microsatellite, the third repeat, when present, is AAC instead of CAC; ^b^ Interrupted microsatellite, in the first TGA microsatellite, the fourth TGA repeat is a TGC.

**Table S2.** Primers used to amplify the sequences studied.

| Gene | Name | Sequence (5′–3′) | References |
| --- | --- | --- | --- |
| *mtDNA* |  |  |  |
| *cox*1 | cytcF | GTTCTTTGGTCATCCAGAAGT | Yang et al. (2008) |
|  | cytcR | ACGACGTAGTAGGTGTCATGF |  |
| *cyt*b | Trich-cob-F1 | CAATCCATTAGGTACACACTCAC | Dunams-Morel et al. (2012) |
|  | Trich-cob-R3 | TAAGTAAGATTTCAATGGCG |
| *Nuclear* |  |  |  |
| ESV | ESVF | GTTCCATGTGAACAGCAGT | Zarlenga et al. (1999) |
|  | ESVR | CGAAAACATACGACAACTGC |  |
| 5S ISR | ISRF | GCGAATTCTTGGATCGGAGACGGCCTG | Rombout et al. (2001) |
|  | ISRR | GCTCTAGACGAGATGTCGTGCTTTCAACG |  |
| ITS1 | ITS1F | GGCTTCGCGCCGGGAAATTTC | Erster et al. (2016) |
|  | ITS1R | TTTAAACCTGATGCACAACAC |  |
| 18S | 18S1F | AAGCTTGCTTGTCTCAAAGATTAAGCC | This study |
|  | 18S1R | AATTGTTCATCTTGCTGCGATCCAA |  |
|  | 18S2F | ATAATGATTGAAAGGAACAGACGGGG |  |
|  | 18S2R | GATCCTTCCGAGTTCACCTACG |  |

**Table S3.** Chinese *Trichinella spiralis* isolates and other related *Trichinella* species included in the molecular analysis, and accession numbers of the corresponding individual sequence. Asterisks indicate sequences newly reported in this study.

| **Sample (ISS code)** | **Accession number** | | | | | |
| --- | --- | --- | --- | --- | --- | --- |
|  | *cox1* | *cyt*b | 5S ISR | ESV | ITS1 | 18S |
| HLJ-hb | MH289517* | MH289523* | MH289505* | MH289529* | MH289535* | MH289511* |
| HN-ny | MH289518* | MH289524* | MH289506* | MH289530* | MH289536* | MH289512* |
| YN-dl | MH289519* | MH289525* | MH289507* | MH289531* | MH289537* | MH289513* |
| HB-xf | MH289520* | MH289526* | MH289508* | MH289532* | MH289538* | MH289514* |
| GX-td | MH289521* | MH289527* | MH289509* | MH289533* | MH289539* | MH289515* |
| YN-dl | MH289522* | MH289528* | MH289510* | MH289534* | MH289540* | MH289516* |
| *T. spiralis* (ISS003) | DQ007890 | KM357422 | N/A | N/A | N/A | AY497012 |
| *T. native* (ISS010) | DQ007891 | KM357415 | N/A | N/A | N/A | AY851256 |
| *T. britovi* (ISS005) | DQ007892 | KM357413 | N/A | N/A | N/A | AY851257 |
| *T. pseudospiralis* (ISS013) | DQ007893 | KM357408 | N/A | N/A | N/A | AY851258 |
| *T. murrelli* (ISS035) | DQ007894 | KM357414 | N/A | N/A | N/A | AY851259 |
| T6 (ISS040) | DQ007895 | KM357418 | N/A | N/A | N/A | AY851260 |
| *T. nelson* (ISS029) | DQ007896 | KM357416 | N/A | N/A | N/A | AY851261 |
| T8 (ISS124) | DQ007897 | KM357419 | N/A | N/A | N/A | AY851262 |
| T9 (ISS409) | DQ007898 | KM357420 | N/A | N/A | N/A | N/A |
| *T. papuae* (ISS572) | DQ007899 | KM357417 | N/A | N/A | N/A | AY851263 |
| *T. zimbabwensis* (ISS1029) | DQ007900 | KM357421 | N/A | N/A | N/A | AY851264 |
| *T. patagoniensis* (ISS2496) | KM357412 | KM357412 | N/A | N/A | N/A | N/A |

**Primer References**

Dunams-Morel DB, Reichard MV, Torretti L, Zarlenga DS, Rosenthal BM (2012) Discernible but limited introgression has occurred where *Trichinella nativa* and the T6 genotype occur in sympatry. Infect Genet Evol 12:530–538

Erster O, Roth A, King R, Markovics A (2016) Molecular characterization of *Trichinella* species from wild animals in Israel. Vet Parasitol 231:128–131

La Rosa G, Marucci G, Rosenthal BM, Pozio E (2012) Development of a single larva microsatellite analysis to investigate the population structure of *Trichinella spiralis*. Infect Genet Evol 12:369–376

Rombout YB, Bosch S, van Der Giessen JW (2001) Detection and identification of eight *Trichinella* genotypes by reverse line blot hybridization. J Clin Microbiol 39:642–646

Yang YR, Jian W, Pozio E (2008) Analysis of cytochrome c-oxidase (COI) gene of mitochondrial DNA from the *Trichinella* spp. in China. Parasitol Res 103:1355–1359

Zarlenga DS, Chute MB, Martin A, Kapel CM (1999) A multiplex PCR for unequivocal differentiation of all encapsulated and non-encapsulated genotypes of *Trichinella*. Int J Parasitol 29:1859–1867
